# SOFT TISSUE SARCOMA - SANTA CASA DE SÃO PAULO EXPERIENCE FROM 2006 TO 2019

**DOI:** 10.1590/1413-785220233103e263799

**Published:** 2023-07-17

**Authors:** BRUNA BUSCHARINO, ANDERSON RODRIGUES DOS SANTOS, DANTE GALVANESE AMATO NETO, MURILO ALEXANDRE, EDUARDO SADAO YONAMINE, PATRICIA MARIA DE MORAES BARROS FUCS

**Affiliations:** 1 Santa Casa de Misericórdia de São Paulo, Departamento de Ortopedia e Traumatologia, São Paulo, SP, Brazil.

**Keywords:** Soft Tissue Neoplasms, Therapy, Soft Tissue, Epidemiology, Neoplasias de Tecidos Moles, Terapia de Tecidos Moles, Epidemiologia

## Abstract

**Objective:**

To conduct an epidemiologic review, analyzing treatment, evolution, and survival of soft tissue sarcomas.

**Methods:**

Retrospective study based on medical records of patient with STS treated by the Orthopedic Oncology Group at the Santa Casa de São Paulo, from 2006 to 2019. Data from 121 patients were analyzed according to age, sex, histological type, tumor location, treatment, previous surgery in a non-specialized service, local recurrences, lung metastases, and survival analysis.

**Results:**

The most frequent location was the thigh. Patients who underwent surgery with a non-specialized group had higher rates of local recurrence and those with pulmonary metastasis had a lower survival rate.

**Conclusion:**

STS can occur at any age and the prevalence of the histological type depends on the patients’ age group. ***Level of Evidence II, Prognostic Study.***

## RESUMO

**Objetivo:**

Conduzir uma avaliação epidemiológica analisando tratamento, evolução e sobrevida dos sarcomas de partes moles (SPMs).

**Métodos:**

Estudo retrospectivo de prontuários de pacientes com SPM tratados pelo Grupo de Oncologia Ortopédica da Santa Casa de Misericórdia de São Paulo, no período de 2006 a 2019. Foram analisados os dados de 121 pacientes referentes a idade, sexo, tipo histológico, localização do tumor, tratamento, cirurgia prévia em serviço não especializado, presença de recidivas, metástases pulmonares e análise de sobrevida.

**Resultados:**

A localização mais frequente foi a coxa. Verificou-se que pacientes que realizaram cirurgia com grupo não especializado tiveram maiores índices de recidiva local, e aqueles com metástase pulmonar tiveram menor sobrevida.

**Conclusão:**

Os SPMs podem ocorrer em qualquer idade, e a prevalência do tipo histológico depende da faixa etária dos pacientes. **Nível de Evidência II, Estudo Prognóstico.**

## INTRODUCTION

Soft Tissue Sarcomas (STS) are uncommon tumors,^1^ represent less than 1% of all malignant tumors in adults, and have a great histological diversity, with more than 50 histological subtypes based on the tumor lineage. ^(^ ^2^ ^)-(^ ^7^ STS rarity and diversity has hindered its study. However, collaborative studies with the formation of large databases and tissues have currently increased the understanding of this group of diseases. ^(^ ^2^ ^),(^ ^8^

Similar to other rare and serious diseases, early diagnosis and access to specialized services directly affects the prognosis, which invariably leads to errors and delays in diagnosis^2^ ^),(^ ^3^ Thus, the best results occur in reference centers. ^(^ ^2^ ^),(^ ^3^

Frequently, STS presents themselves initially as slightly painful tumors, delaying and hindering the diagnosis. ^(^ ^4^ They can occur at any age and anatomical location, with a predominance of 75% in the limbs and especially in the thigh. ^(^ ^4^ As in other malignant neoplasms, STS incidence increases with advancing age, especially after 65 years old. ^(^ ^3^

At diagnosis, 10% of patients already present metastases, mainly lung lesions. ^(^ ^3^

Prognostic factors related to STS are histological grade, tumor size, and microscopic margin after resection. ^(^ ^5^ ^),(^ ^6^ A better understanding of the behavior of these tumors may result in better surgical treatment and the development of new adjuvant therapies. ^(^ ^7^ ^),(^ ^9^ ^)-(^ ^12^

We believe that the initial step for the development of new treatments is the understanding of the behavior of a disease by an epidemiological study. The rarity and diversity in the behavior of STS hinder the publication of new studies. Thus, we aimed to study our cases and publish our outcomes.

Therefore, this study aimed to show the epidemiological data of patients with soft tissues sarcoma in the musculoskeletal tumors service of the Departamento de Ortopedia e Traumatologia of the Hospital Geral da Santa Casa de Misericórdia de São Paulo.

## METHODS

This is a retrospective study, carried out by the analysis of medical records of patients diagnosed with STS who were treated by the Orthopedic Oncology Group of Santa Casa de São Paulo, from January 2006 to December 2019 (Approval Protocol of the CAAE Ethics Committee: 76547317.6.0000.5479).

Data were collected by an evaluation instrument (Appendix 1), including the following information: sex, age, histological type of tumor, tumor size at the time of diagnosis, location, type of treatment performed (surgery, chemotherapy, radiotherapy), previous surgery with another team, presence of lung metastases, local recurrence during follow-up, and date of death.

During the study period, 169 patients with biopsy-confirmed STS diagnosis were treated, 48 patients were excluded due to incomplete information in the medical record.

The statistical analysis was performed with 121 patients in two stages: descriptive analysis and inference.

In the inference, contingency tables were constructed to study the association between qualitative variables. Chi-Square and Fisher’s Exact tests were used when convenient.

In the survival analysis, the Kaplan-Meier method was used to study the relationship between factors and time.

In all tests, a 5% significance level was adopted.

## RESULTS

**Figure 1:**
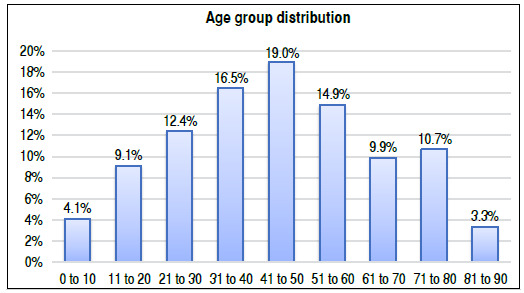
Distribution of the cases according to age group, of the 121 patients treated by the orthopedic oncology group from 2006 to 2019.

In our sample, we observed a minimum age of 6 months and a maximum age of 85 years, mean age of 45.4 years (± 20.2 SD). The incidence peak was in the age group of 41 to 50 years (Figure 1). Females presented a small predominance of 52% (n = 63), and males composed 48% (n = 58) of the sample.

The most frequent histological types in the extremities were synovial sarcoma with 34 cases (28.1%), undifferentiated sarcoma with 20 cases (16.5%), and liposarcoma with 20 cases (16.5). Table 1 shows the remaining histological types.

**Table 1:** Distribution of the histological type of patients treated by the orthopedic oncology group from 2006 to 2019.

Histological type	(%)*	(n)
Synovial sarcoma	28.1%	34
Liposarcoma	16.5%	20
Undifferentiated sarcoma	16.5%	20
Leiomyosarcoma	8.3%	10
Neurofibrosarcoma	8.3%	10
Myxofibrosarcoma	5.8%	7
Rhabdomyosarcoma	4.1%	5
Fibromyxoid sarcoma	4.1%	5
Dermatofibrosarcoma	1.7%	2
Infantile fibrosarcoma	1.7%	2
Solitary fibrous tumor	1.7%	2
Angiosarcoma	0.8%	1
Epithelioid sarcoma	0.8%	1
Myxoinflammatory fibroblastic sarcoma	0.8%	1
Myofibroblastic sarcoma	0.8%	1

* Percentages regarding the 121 patients in the sample.

The most frequent location of STS was the thigh (n = 59;48.8%), followed by the arm (n = 14; 11.6%), the leg (n = 12; 9.9%), and the pelvic girdle (n = 9; 7.4%). The other locations are distributed by anatomical region (Table 2).

**Table 2:** Distribution according to tumor location of patients treated by the orthopedic oncology group from 2006 to 2019.

Location	(%)*	(n)
Thigh	48.8%	59
Arm	11.6%	14
Leg	9.9%	12
Pelvic girdle	7.4%	9
Foot	5.8%	7
Elbow	3.3%	4
Forearm	2.5%	3
Pectoral girdle	2.5%	3
Back	2.5%	3
Knee	2.5%	3
Hand	1.7%	2
Flank	0.8%	1
Chest	0.8%	1

* Percentages regarding the 121 patients in the sample.

Regarding tumor size, patients were divided into groups with tumors larger, and smaller than 5 cm, 113 (93%) and eight (7%) patients, respectively.

The treatment for patients diagnosed with STS was surgical intervention (87.6%). In total, 88 patients (67.8%) underwent tumor resection surgery with limb preservation and 24 (19.8%) underwent amputation surgery. Only 15 (12.4%) of the patients did not undergo surgical intervention due to clinical complications during chemotherapy or radiotherapy, advanced stage of the disease, or refusing the surgical treatment proposed.

In total, 72 (59.5%) patients underwent chemotherapy.

In total, 57 (47.1%) patients underwent complementary treatment with local radiotherapy, mostly, 53 (43.8%), in the postoperative period. In our study, 24 (22.4%) patients evolved with local recurrence of the tumor. Those who underwent previous surgery in another service and/or with a non-specialized group experienced recurrence in 40.9% (P < 0.005) (Table 3).

**Table 3:** Distribution according to tumor location of patients treated by the orthopedic oncology group from 2006 to 2019.

		Without recurrence		With recurrence		Total		***p*-value**
		N	(%)*	N	(%)*	N	(%)*	
Previous surgery	No	84	84.8	13	59.1	97	80.2	0.006
	Yes	15	15.2	9	40.9	24	19.8	

* Percentages regarding the 121 patients of the sample.

**Figure 2:**
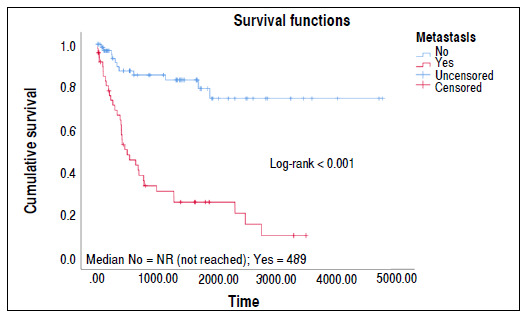
Kaplan-Meier survival curve for lung metastases.

During follow-up, 49 (40.5%) developed pulmonary metastasis. In total, 46 patients (38%) died during follow-up. Patients with lung metastasis had lower survival (Figure 2).

## DISCUSSION

In our sample, STS is not prevalent regarding sex, as is the Memorial Sloan Kettering Cancer Center and the rest of the literature. ^(^ ^6^ ^),(^ ^13^ ^)-(^ ^16^

The predominant age group ranged from 31 to 60 years, considering that they were treated in a general hospital that assists adults and children. Corroborating epidemiological data in the literature, the older the patients the higher the incidence of STS. ^(^ ^6^ ^),(^ ^15^ ^)-(^ ^17^

We found a difference between the predominance of the histological type in patients treated at the Santa Casa de São Paulo compared to the literature. Synovial sarcoma is more prevalent in young patients, as well as the profile of patients evaluated in the general hospital within the department of orthopedics and traumatology, which assists younger patients. Moreover, these cases relate only to extremity tumors, different from the ones of the Hospital Sloan Kettering Memorial, which also includes abdominal, retroperitoneal, and thoracic tumors. The work of the Mayo Clinic, also in the United States, reveals a predominance of Liposarcoma in their cases, however, it includes cases in the retroperitoneum and abdomen. ^(^ ^6^ ^),(^ ^15^ ^)-(^ ^19^ We also observed that the predominance of the histological type is associated with the predominant age in each service.

The most frequent location of soft tissue sarcomas of the extremities was in the thigh, regardless of the predominance of age, sex, or histological type. Studies from the literature also reported the thigh as the most affected location among the STS of the extremities. ^(^ ^2^ ^),(^ ^6^ ^),(^ ^16^ ^),(^ ^17^

Surgical treatment was chosen, an expected result since the treatment of STS is predominantly surgical. Most patients were subjected to an attempt to preserve the limb with tumor resection. ^(^ ^18^ Radiotherapy as treatment was associated with surgery almost exclusively in the adjuvant form. The Orthopedic Oncology Group indicates radiotherapy treatment for resection with a borderline oncologic margin, for histological types that have a low response to chemotherapy, as a complementary treatment for patients with high-grade sarcoma. This is a preference of the team regarding neoadjuvant radiotherapy to avoid complications with surgical wound. ^(^ ^6^ ^),(^ ^15^ ^)-(^ ^17^ ^),(^ ^20^

Some patients with STS were biopsied or initially treated in another service before being referred to a specialized service, 15 to 20% of the patients according to the literature, and with the data from our service (19%).^6^ ^),(^ ^15^ ^)-(^ ^17^ Local recurrence for these patients was statistically significant (p ≤ 0.05) and much higher (40.9%) than for the rest of the sample.

Our global local recurrence was 22.8%, as found in the literature (20-25%).^6^ ^),(^ ^15^ ^)-(^ ^17^

Almost half patients (40.5%) developed pulmonary metastases at some point during treatment. This finding is associated with decreased patient survival. ^(^ ^6^ ^),(^ ^14^

Considering the nature of this study, some factors hampered data collection, namely: incorrect filling of medical records; old medical records without digitized version and with loss of information; inaccurate dates of examinations and deaths; loss of old pathological and anatomical results; difficulty contacting patients and their families; and incomplete data in medical records.

## CONCLUSIONS

The predominant age group of patients with STS in our service ranged from 41 to 50 years. The most prevalent histological types were Synovial Sarcoma, followed by Undifferentiated Sarcoma and Liposarcoma. The most affected location was the thigh. The treatment was predominantly surgical with limb preservation. Those who have had previous surgery in another service have higher rates of tumor recurrence. Patients with lung metastasis have lower survival.

## APPENDIX 1

**Soft Tissue:** Sarcoma Evaluation Protocol (Appendix 1)

Register:				
Name:				
Age:		Phone number:		
Sex:		Histological type:		
( ) M	( ) F			
Location:				
( ) Pectoral girdle		( ) Pelvic girdle		
( ) Arm		( ) Thigh		
( ) Forearm		( ) Knee		
( ) Hand		( ) Leg		
( ) Elbow		( ) Foot		
( ) Wrist		( ) Ankle		

Chemotherapy:		If yes:		
( ) Yes	( ) No	( ) I do not know	( ) Adjuvant	( ) Neo adjuvant

Radiotherapy:		If yes:		
( ) Yes	( ) No	( ) I do not know	( ) Adjuvant	( ) Neo adjuvant

Surgery:		If yes:		
( ) Yes	( ) No	( ) I do not know	( ) Intralesional resection	
			( ) Marginal resection	
Date:			( ) Wide resection	
			( ) Radical resection - amputation	
			( ) I do not know	
Tumor size:		( ) ≤ 5 cm	( ) ≥ 5 cm	( ) I do not know
Presence of metastasis:		( ) Yes	( ) No	( ) I do not know
Previous surgery by another service:		( ) Yes	( ) No	( ) I do not know
Local recurrence:		( ) Yes	( ) No	( ) I do not know
Date of diagnosis:		Date of last consultation:		
Death:		( ) Yes	( ) No	( ) I do not know
Date:				
